# Pathological effects and immune modulation in host during Tilapia Parvovirus (TiPV) outbreak in cage and wetland Tilapia farms

**DOI:** 10.1038/s41598-024-79089-5

**Published:** 2024-11-20

**Authors:** Basanta Kumar Das, Vikash Kumar, Suvra Roy, Ramesh Chandra Malick, Kampan Bisai, Asim Kumar Jana, Souvik Dhar

**Affiliations:** Aquatic Environmental Biotechnology Division, ICAR-Central Inland Fisheries Research Institute, Barrackpore, 700120 Kolkata India

**Keywords:** Disease outbreak, Tilapia, Tilapia Parvovirus, Pathological condition, Immune response, Viral infection, Immunology

## Abstract

Viral diseases arising in farmed fish are an ongoing challenge to the aquaculture industry, causing severe mortality and economic losses. Recently, there has been a spike in the incidence of a viral disease caused by Tilapia Parvovirus (TiPV) inflicts irreparable damage, and large-scale fish kills in the farmed tilapia species. We investigated a case of disease outbreak and severe mortality in cage and wetland farms of tilapia in West Bengal and Odisha, India. The symptomatic fish showed clinical signs, including hemorrhage, discoloration, ulcer, and redness in the body surfaces. Further analysis revealed that Tilapia Parvovirus was associated (validated by PCR, phylogenetic analysis, and cell line assay) with the infection and mortality of tilapia. The virus was detected in gill, heart, spleen, liver, and kidney samples collected from apparently healthy (asymptomatic) and symptomatic tilapia samples from cage and wetland farms. At the same time, negative results were found in the brain and skin tissue samples. The histological analysis revealed that TiPV induces severe damage invariably in almost all studied tissue, including the liver, kidney, spleen, gill, heart, and brain of tilapia samples. The viral quantification analysis showed that the viral genome was higher in the liver, spleen, and heart than in the tilapia samples’ gill, kidney, or brain tissue. Furthermore, the study indicated that TiPV infection has a significant effect on the health of tilapia. The tilapia exhibited an immune reactivity toward TiPV infection (upregulation of chemokine receptors, *CRs* and interleukin 1β, *IL-1β*), the majority of the studied immune genes (interleukin 8, *IL-8*; Toll-like receptors 7, *TLR7*; tumour necrosis factor α, *TNF-α*; major histocompatibility complex II, *MHC II* and nuclear factor kappa B, *NF-kB*) were significantly downregulated in the kidney, spleen and liver tissue samples of symptomatic tilapia. Further, the in vivo challenge assay confirms that the isolated TiPV is a novel parvovirus pathogen that causes massive mortality in tilapia. The lessons learned from the first cellular and molecular description associated with TiPV epidemiology from wetland and cage farms of tilapia could be critical to developing the current state of the tilapia farming industry. Additionally, a holistic approach is needed to develop management measures to control the virulence and risk factors of TiPV.

**Supplementary Information:**

The online version contains supplementary material available at 10.1038/s41598-024-79089-5.

## Introduction

Tilapia are the most popular farmed aquatic animals, native to Africa and being cultured on a large scale in more than 145 countries worldwide, contributing significantly to millions of people’s socioeconomic development and nutritional security^1,2^. Tilapia has become a critical internationally traded aquaculture commodity; production of cultured tilapia increased rapidly, making it the second-most important group of farmed aquatic animals by quantity^3,4^. The annual worldwide production of tilapia stands at roughly 6.5 million tons, with a current market value surpassing $11 billion U.S. dollars (USD)^5^. Furthermore, over the past 30 years, there has been significant growth in tilapia aquaculture due to the increasing global demand for its consumption^6^. Asia dominates global production, with 72% of the total output, primarily contributed by China and Southeast Asia^7,8^.

The widespread movement of live fish and their products across national borders, together with the global development of tilapia farming, may contribute to the establishment and spread of new emerging diseases that might impact aquaculture^9–11^. Many fungi, bacteria, and parasite diseases have been described to affect China tilapia aquaculture^12,13^. Several new viral pathogens have recently created havoc in tilapia farming, causing massive mortality and economic losses. During August-September of 2023, a case of mortality and massive losses was reported in cage and wetland tilapia farms in two states of India. Routine monitoring of known bacterial pathogens and parasites did not reveal abnormalities or resolve the enigma. Later, attempts were made to identify the known bacterial and viral agents of tilapia, such as viral nervous necrosis (VNN), herpes-like tilapia larvae encephalitis virus (TLEV), Tilapia Lake virus (TiLV), iridovirus and betanodavirus were unsuccessful^4,14–17^. These results led us to suspect an emerging viral pathogen, i.e., Tilapia Parvovirus (TiPV), recently reported from Nile tilapia in China, India and red hybrid tilapia in Thailand^8,18–21^.

In 2019, the novel *Tilapia Parvovirus* (TiPV), tentatively named *chapparvovirus*, was first characterized in China^22^. TiPV is a nonenveloped, small, single-stranded DNA (ssDNA) virus containing ~ 4000–6000 long nucleotides with ORF1 and ORF2 as two main open reading frames (ORFs). While ORF1 is responsible for encoding non-structural protein 1 (NS1), ORF2 encodes the structural protein 1 (VP1). Since 2015, TiPV has been linked to deadly outbreaks within the Chinese tilapia aquaculture sector^8^. Initially, the virus was thought to be non-infectious as it was identified from the intestinal samples of healthy tilapia and fecal samples from tilapia-fed crocodiles. In 2019, Thailand reported the first incidence of TiPV beyond China’s borders, specifically in red tilapia farming^6^. Subsequently, it was observed that the virus can be lethal to tilapia and cause mortalities up to 90%. In China, an outbreak of TiPV resulted in the mortality of tilapia of 60–70% in natural conditions. In Thailand, the mortality percentage was recorded as 50–75% in all age groups of tilapia. The viral signature of TiPV was detected in various organs of tilapia, including eyes, brain, gills, heart, liver, anterior kidney, intestine, dorsal muscle, spleen, and mucus, with the highest load of TiPV being observed in the anterior kidney and spleen^6,18,23^. TiPV infection can lead to a range of symptoms, including lethargy, reduced feeding, discoloration, cutaneous hemorrhages, exophthalmia, and severe ocular lesions, and abnormal swimming behaviors. In several instances, coinfection of TiPV was reported with TiLV and other bacterial pathogens with polymicrobial infections in farm-raised tilapia in floating cages and wetland aquaculture in different parts of the world, including Thailand, India and China, thus indicating the need for further investigations of disease associated with TiPV and its related pathobiology to determine the impacts of TiPV on tilapia farming^18^.

This study aimed to isolate the TiPV and characterize the host-pathogen response during the TiPV disease outbreak. In this study, we isolated the TiPV from healthy and diseased fish and further characterized it using PCR, gene sequence analysis, and cell line assay. Further investigations were carried out to evaluate the cellular pathological changes during infection and quantify viral load and immune gene expression in different tissue samples of tilapia during TiPV infection. This is the first comprehensive study in which we provided a complete profile of cellular and molecular changes during parvovirus infection in fish.

## Materials and methods

### Collection of fish samples

A case of severe infection and mortality in tilapia cage and wetland culture facilities from different sizes of tilapia (Length = 120.4–245 mm, Weight = 12.51–750 g) from two geographical locations, viz., West Bengal and Orissa in India, were reported during August-September, 2023. The cumulative mortality of tilapia ranged from 60 to 70% in wetland farms and 80–90% in cages. The aquaculture farms in West Bengal and Orissa have been stocked with tilapia along with Indian Major Carps (IMCs), minor carp, and exotic carp. In contrast, monosex tilapia (*Oreochromis niloticus*) was cultured in Orissa’s cage farms. The symptomatic fish showed clinical signs, including hemorrhages, ulcers, discoloration, and redness in fins and all over the body surface, and internally with marked intestinal tract enlargement with fluid accumulation were collected from the aquaculture farm and transferred to ICAR-Central Inland Fisheries Research Institute, Kolkata Fish Pathology Lab for the etiological agents screening. A total of 50 asymptomatic and symptomatic fish from each cage and wetland farm were collected for analysis. Fresh smears of skin and gills were examined under a microscope in the lab to check for ectoparasites. The fish were then euthanized by an overdose of MS222 (Sigma-Aldrich), and post-mortem exams were carried out to document various clinical signs in the internal organs. An attempt was made to isolate bacteria from the liver, kidneys, and blood samples of three symptomatic fish under aseptic conditions. The collected samples were homogenized aseptically in 8 mL of distilled water for 20–30 s at room temperature (RT). Afterward, using sterile 1X PBS, the homogenate was serially diluted, and 100 µL of each dilution was spread onto TSA Petri dishes and incubated at 28 °C for 18–20 h. The plates with 30–300 CFU/mL were used to count the colonies number at specific dilutions and calculate the abundance of bacteria in the symptomatic and control fish. Afterward, based on uniqueness in morphology, size, and color, the colony was picked from TSA plates and cultured overnight in Tryptone soya broth (TSB) at 28 °C for further characterization (molecular and virulence through in vivo challenge assay). For molecular studies, different tissues from symptomatic and asymptomatic tilapia tissue samples were collected in RNA later. Further, internal organs viz., kidney, liver, and spleen tissues were fixed in neutral buffered formalin (10%) for histopathological analysis. The post-mortem and clinical examinations were conducted according to established procedures. The Institutional Animal Ethics Committee, ICAR-CIFRI, Kolkata, India, has approved the animal utilization protocol for the experimental setup. The study is reported following the ARRIVE guidelines.

### DNA isolation and PCR amplification

Following the manufacturer’s instructions, a DNeasy Blood and Tissue kit (Qiagen, Germany) was used to extract total genomic DNA from tissue samples of moribund fish. For the assay, 15 asymptomatic and symptomatic fish from each cage and wetland farm were pooled with five fish/replicate in three replicates. A UV-visible spectrophotometer was used to measure the concentration of DNA. The isolated DNA was examined using a 1.8% agarose gel, and Nano-drop (Eppendorf, Germany) was used to assess the DNA quality. The PCR amplification was performed using the primer of Tilapia Parvovirus (Liu et al.^8^) (Table 1). The final volume of the PCR reaction mixture maintained at 50 µl consists of 1 µl of 10 mM dNTP, 100 ng of isolated genomic DNA., one µl of 50 mM MgCl_2_, one µl of 10 pmol of each primer, 1 µl of Taq DNA Polymerase and five µl of 10× PCR buffer (Sigma, USA). A primary denaturation step at 95 °C for 5 min was followed by 35 cycles of denaturation at 94 °C for 30 s, annealing at 55 °C for 30 s, extension at 72 °C for 60 s, and final extension at 72 °C for 10 min in the PCR process. Electrophoresis was used to analyze the amplified products using 1.8% agarose gels and ethidium bromide staining. The anticipated 534 bp PCR products showed positive Tilapia parvovirus results.

**Table 1:** Primers used in the study.

Gene	Primer Sequence 5’ to 3’	Annealing temperature	References
*TiPV-F*	GAGATGGTGTGAAAATGAACGGG	60 °C	^8^
*TiPV-R*	CTATCTCCTCGTTGCTCGGTGTATC		
*TiPV-Fq*	GCACCACAGCTGAGTACAAC	56 °C	
*TiPV-Rq*	AACTGCTCGGCTATCTCCTC		
*β-Actin-F*	CAGCAAGCAGGAGTACGATGAG	60 °C	^61^
*β-Actin-R*	TGTGTGGTGTGTGGTTGTTTTG		
*IL8-F*	GCACTGCCGCTGCATTAAG	60 °C	
*IL8-R*	GCAGTGGGAGTTGGGAAGAA		
*TLR7-F*	TCAGCAGGGTGAGAGCATAC	60 °C	
*TLR7-R*	ACATATCCCAGCCGTAGAGG		
*MHC-II-F*	TGGCCCTGACTGAACCACTG	60 °C	
*MHC-II-R*	TCAGACCCACGCCACAGAAC		
*NFκB-F*	AACGACGGTGATGACAACGAC	60 °C	
*NFκB-R*	AAATTCAGGCTCCACACTGACC		
*CRs- F*	ACAGAGCCGATCTTGGGTTACTTG	60 °C	
*CRs- R*	TGAAGGAGAGGCGGTGGATGTTAT		
*IL1β -F*	TGCACTGTCACTGACAGCCAA	60 °C	
*IL1β -R*	ATGTTCAGGTGCACTATGCGG		
*TNF-α -F*	CCAGAAGCACTAAAGGCGAAGA	60 °C	
*TNF-α -R*	CCTTGGCTTTGCTGCTGATC		

### Phylogenetic analysis of Tilapia Parvovirus

The amplified gene was sequenced in both directions using an ABI 373xl capillary sequencer (Applied Biosystem, Foster City, CA). DNA Baser 7.0 was used to match the forward and reverse sequences, resulting in a contig. The sequence was then uploaded to GeneBank and NCBI. The NCBI-BLAST tool was then used to compare the approximately 534 bp viral gene sequence with other sequences that were stored in GeneBank. The gene sequence of the virus was compared to the gene sequences of the most commonly detected Tilapia parvovirus that was obtained from the NCBI gene bank. Using the neighbor-joining method^25^, a phylogenetic tree was generated using MEGA 11.0^24^ and designed with iTOL v4 (Interactive Tree of Life) software^26^.

### Fish cell line infection

Using the standard protocol, the tissue homogenate for infecting the cell lines was prepared from nine symptomatic tilapia spleen samples pooled with three fish/replicate in three replicates. In brief, sterile Leibovitz’s-15 (L-15) media supplemented with a 1× concentration of an antibiotic-antimycotic solution (Life Technologies, Darmstadt, Germany) was used to homogenize pooled tissues. The homogenized tissues were frozen and thawed three times to release the virus particles from the infected cells. The homogenate was centrifuged for 15 min at 4 °C at 6500 g, and the supernatant was filtered using a Millipore (Carrigtwohill, Ireland) 0.22 μm filter. A confluent monolayer of the DRG cell line derived from *Danio rerio* (NRFC, ICAR-NBFGR) was inoculated with the filtrate (500 µl) and placed in 25 cm^2^ flasks (Nunc, Roskilde, Denmark) that were kept at 28 °C for incubation. Filtrate was replaced with 500 µl of maintenance medium in the control flasks. The filtrate was discarded after one hour of adsorption at room temperature, and each flask was then filled with five millilitres of a maintenance medium (L-15 media with 2% FBS). For seven days post-inoculation, cells were observed daily under an inverted microscope (Nikon, Japan) for any cytopathic effect (CPE). In contrast, the cell pellets were collected in molecular-grade alcohol and stored at – 80 °C for PCR. Subsequently, total genomic DNA was extracted from cell line samples using a DNeasy Blood and Tissue kit; as discussed above, PCR amplification was performed to confirm the presence of Tilapia Parvovirus.

### Histological analysis

The asymptomatic and symptomatic tilapia (5 fish each from cage and wetland farms) were anesthetized with clove oil (50 µL of Clove oil/ L water), and gill, liver, kidney, brain, spleen, and heart samples were collected and fixed in 10% NBF (neutral buffered formalin). Subsequently, the fixed tissues were washed and were cut into 1–2 mm pieces. The samples were dehydrated using various ethanol gradients and treated with xylene (a clearing agent). After an impregnation procedure, the treated tissues were embedded in paraffin using the Leica EG 1140 H embedding machine. Haematoxylin and eosin were used to stain the paraffin-embedded tissue after it was sectioned using a microtome to maintain a five µm thickness (Kumar et al. 2014, 2022). Afterward, a light microscope was used to examine the processed sections for cellular alterations.

### Quantification of TiPV in different tissue

A real-time PCR assay detected and quantified TiPV in symptomatic tilapia gill, heart, spleen, liver, kidney, and brain samples. Samples were collected from nine symptomatic and non-symptomatic tilapia tissues pooled with three fish/replicate in three replicates. To estimate viral load at different point intervals post-infection, the gill, heart, spleen, liver, and kidney samples were collected at 1–6 days following TiPV infection (0.5 ml viral supernatant from the second passage administered intraperitoneally at an amount of 10^4.0^ TCID_50_/ml) for quantitative real-time PCR analysis. Primarily, the previously described techniques were utilized to perform quantitative analyses and real-time PCR processes^22^. The assay was carried out in a 20 µl reaction volume using the following mixture: 2×SYBR Mix 10 µl, primers 0.25 µl each (TiPV-Fq/Rq), DNA 1 µl, ddH_2_O 8.5 µl and the program was 94 °C for 2 min, followed by 40 cycles of 94 °C for 10 s, 60 °C for 30 s (Table 1). Using qPCR analysis, every sample from the symptomatic group was examined three times. A standard curve was created using serial dilutions of artificial DNA fragments derived from the TiPV gene to ascertain the copy counts of the TiPV DNA. Per the manufacturer’s instructions, the viral genome copies per 1 mg of tissues were computed using the Ct values (Qiagen, Germany).

### RNA extraction and reverse transcription

Using the Trizol^®^ reagent and the manufacturer’s recommended procedure, total RNA was extracted. Briefly, liver, kidney, and spleen tissues were collected from nine asymptomatic and symptomatic tilapia samples pooled with three fish/replicate in three replicates and immediately frozen in liquid nitrogen and stored at – 80 °C. A total of 15 asymptomatic and symptomatic fish from each cage and wetland farm were pooled in three replicates with five fish/replicate. The tissue sample was homogenized aseptically for 15–30 s using 1 ml of cold Trizol^®^ at room temperature, and it was then incubated for 5 min at 20 °C. Following this procedure, the homogenate was combined with 200 µl chloroform and agitated vigorously for 15 min at 20 °C. It was then centrifuged for 10 min at 10,000 rpm. 500 µl of isopropanol was added to a fresh tube containing the upper layer of aqueous material. After being stored at − 20 °C for two hours, the solution was centrifuged for ten minutes at 10,000 rpm. To get rid of any remaining ethanol, the resulting pellet was centrifuged for 10 min at 7000 rpm, cleaned with 75% ethanol, and allowed to air dry for a short while. After that, the RNA pellets were dissolved in 50 µl of sterile water treated with DEPC, and the suspension was kept cold until additional examination. The RNA samples were processed with RNase-free DNAse I (Thermo Scientific, India) to eliminate genomic DNA contamination. The NanoDrop Spectrophotometer (Thermo Scientific, India) was used to measure the absorbance at 260/280 to assess extracted RNA’s purity and concentration (ng µl-1). The integrity of the RNA was then examined on a 2% agarose gel. Thermo Fisher Scientific’s RevertAid™ H-Minus First Strand cDNA Synthesis Kit, purchased in India, was used for reverse transcription following the manufacturer’s instructions. Before being used again, the synthesized cDNA sample was kept at − 20 °C, and PCR assessed its quality.

### Quantitative real-time PCR analysis

The expression of immune-related genes comprising of Interleukins (*IL8*, *IL1β*), Toll-like receptors (*TLR7*), major histocompatibility complex (*MHC-II*), Nuclear factor kappa B (*NF-κB*), chemokine receptors (*CRs*) and Tumour necrosis factor α (*TNF-α*) were quantified and compared using StepOnePlus Systems’ (Applied Biosystems, US) real-time PCR method using a specific pair of primers to the housekeeping gene β-actin (also to check for the integrity of RNA) (Table 1)^27–32^.

For the amplification of the target genes, a total reaction volume of 20 µl was used, comprising 1 µl cDNA (50 ng), 10 µl 2X Maxima SYBR Green/ROX qPCR Master Mix (Thermo Fisher Scientific), 0.5 µl of each particular primer, and 8 µl nuclease-free water. The master mix was made in triplicate for each biological replication of the sample, and a four-step amplification technique was used for RT-qPCR to detect immune-related and housekeeping genes. Initial denaturation is done for 10 min at 95 °C. Amplification and quantification are done for 40 cycles of 15 s at 95 °C, 30 s at 60 °C, and 30 s at 72 °C. The melting curve is set between 55 and 95 °C with a heating rate of 0.10 °C/s, a continuous fluorescence measurement, and a cooling period of 4 °C. A reaction mixture serving as a negative control for each primer set was prepared by leaving out the cDNA template. The target gene expression level was estimated using the 2^−ΔΔCt^ approach (comparative CT method), as described by Livak and Schmittgen^33^ and confirmed using Pfaffl’s^34^ relative standard curve method. A t-test was performed on the log-transformed 2^−ΔΔCT^ data, and *P*-values less than 0.05 were regarded as statistically significant.

### Challenge assay with TiPV

The Organisation for Economic Cooperation and Development (OECD) adhered to the standards for the treatment and care of experimental animals. The Institutional Animal Ethics Committee of the ICAR-Central Inland Fisheries Research Institute in Kolkata, India, approved the animal utilization policy for the experimental setting.

Healthy *Oreochromis niloticus* (length 92.12 ± 3.41 mm and weight 62.46 ± 2.42 gm) were bought from fish hatcheries. The experiment involved fish that showed no outward signs of illness, like ulcers, hemorrhage scale loss, discoloration, or redness on the body surface, but instead looked healthy. Thirty fish were randomly chosen and subjected to a conventional approach to screen for the potential presence of infectious microorganisms^21^. The TiPV was challenged in triplicates by intraperitoneal (IP) injection with 0.5 ml supernatant of the 2nd passage of the virus, which had a titer of 10^4.0^ TCID_50_/ml. In the 2nd experiment, 30 nos of tilapia were challenged intraperitoneally with 50 µl of diseased fish tissues homogenate, filtered through a 0.22 μm filter. While in the control group, fish were injected with 50 µl of PBS solution. After injection, all fish were kept in FRP tanks supplied with aerated water at 28 °C. Clinical signs and mortality were monitored daily, and samples from three moribund or dead fish per group (including controls) were randomly collected and processed to determine the presence of TiPV in the samples.

### Statistical analysis

The survival data were converted using arcsin to meet the conditions for homoscedasticity and normalcy. Using a statistical tool for the social sciences (SPSS) version 24.0, they were put through a one-way analysis of variances (ANOVA) and Duncan’s multiple range test. Results for the gene expressions were represented as fold changes relative to the geometrical mean of two internal control genes (β-actin). The expression level in the control was regarded as 1.0, and thereby, the expression ratio of the treatments was expressed in relation to the control. Analysis for significant differences in expression levels between the control and treatment groups was performed with the single-tailed Student’s t-tests using log-transformed data. The significance level was set at *P* ≤ 0.05.

## Results

### Characterization of pathological condition in Tilapia

**Fig. 1:**
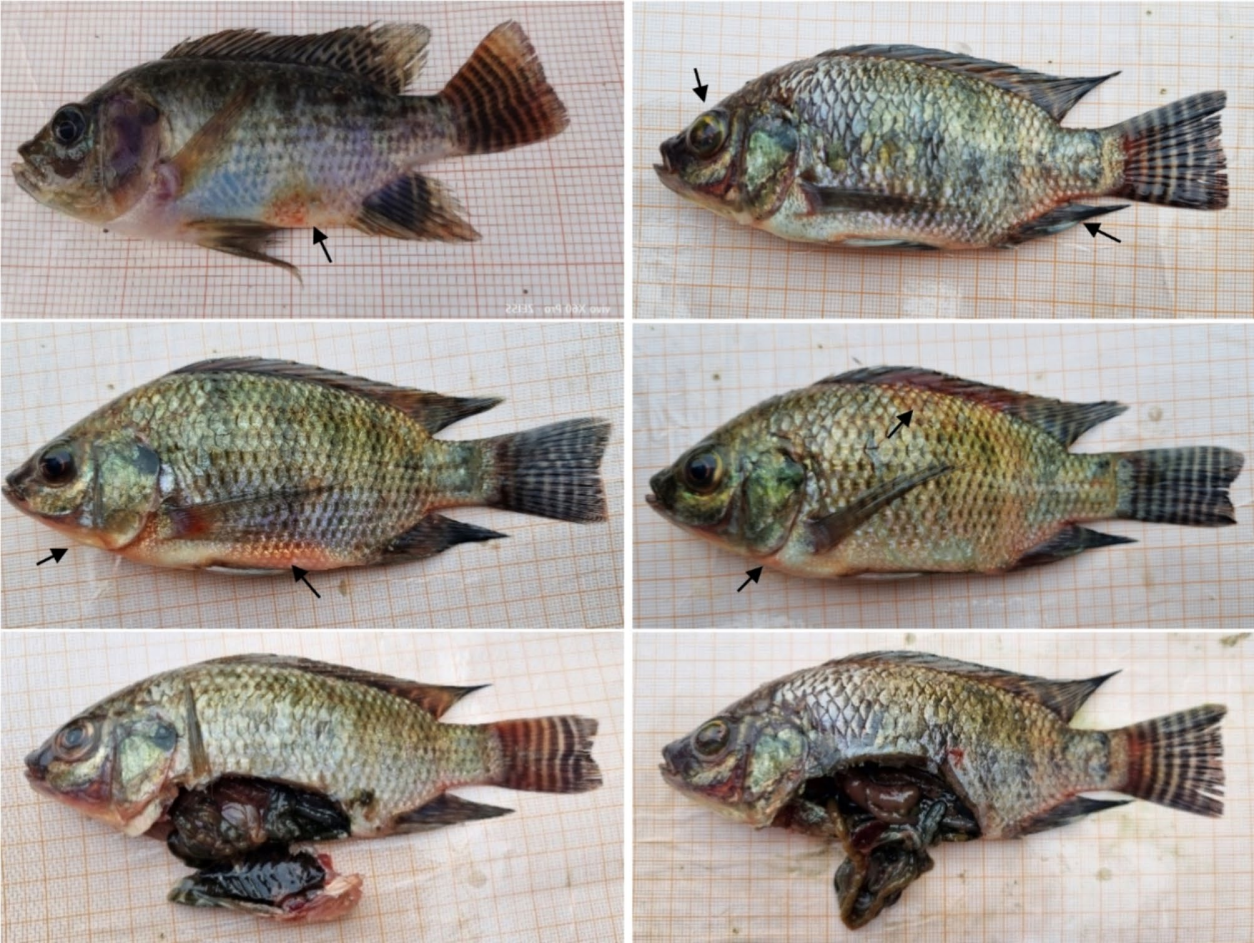
Symptomatic tilapia samples collected from wetland and cage farms. Gross pathological signs include hemorrhages on the lower jaw and anterior abdominal (yellow arrowhead), accompanied by exophthalmos eyes and pronounced ocular lesions (red arrowhead). Later, the post-mortem examinations of tilapia were done to observe possible changes in internal organs (black arrowhead).

Affected fish showed clinical signs, including hemorrhages, ulcers, discoloration, and redness in fins and all over the body surface, and internally with marked intestinal tract enlargement with fluid accumulation (Fig. 1). The cumulative mortality of tilapia ranged from 60 to 70% in wetland farms and 80–90% in cages. The clinical signs recorded in this study coincide with previous works reported for microbial infection in tilapia. Moreover, no clinical signs or gross lesions were observed in cohabiting fishes, including Indian major carp, minor carp, and exotic carp on the same farm. Suspecting a microbial infection, we first tried to isolate potential pathogenic bacteria from symptomatic tilapia samples. Although few non-pathogenic bacteria like *Citrobacter freundii*, *Enterobacter cloacae*, etc., were isolated (based on survival assay and molecular characterization), no pathogenic bacteria, e.g., *Streptococcus* sp., were isolated from the liver, kidneys, and blood samples of symptomatic fish. Later, screening was performed for the possible presence of major tilapia viral pathogens. Results showed that symptomatic tilapia samples were negative for nervous necrosis virus (NNV), infectious pancreatic necrosis virus (IPNV), tilapia larvae encephalitis virus (TLEV), infectious spleen and kidney necrosis virus (ISKNV), the Iridovirus infections, namely Bohle iridovirus (BIV), Lymphocystis disease virus (LCDV), and tilapia lake virus (TiLV).

**Fig. 2:**
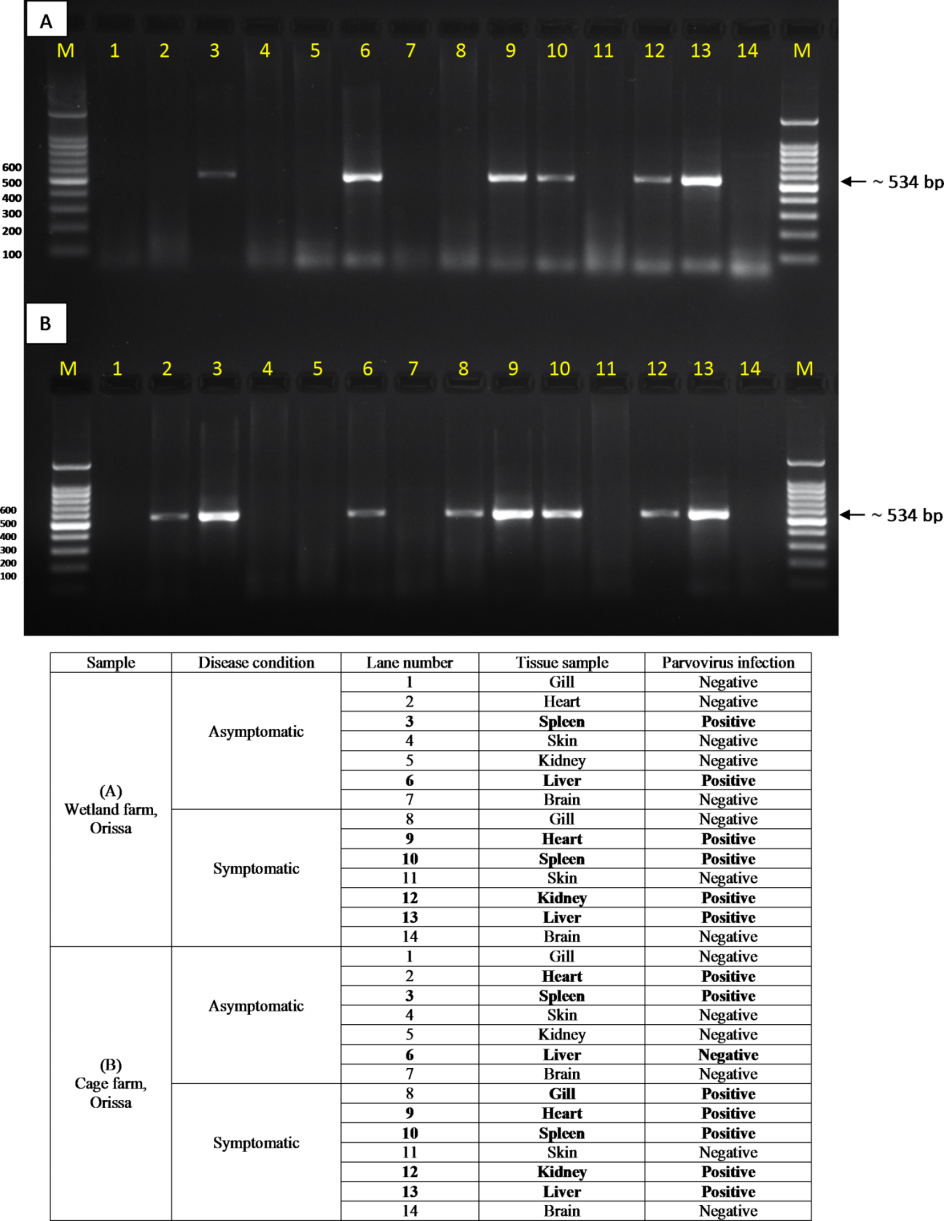

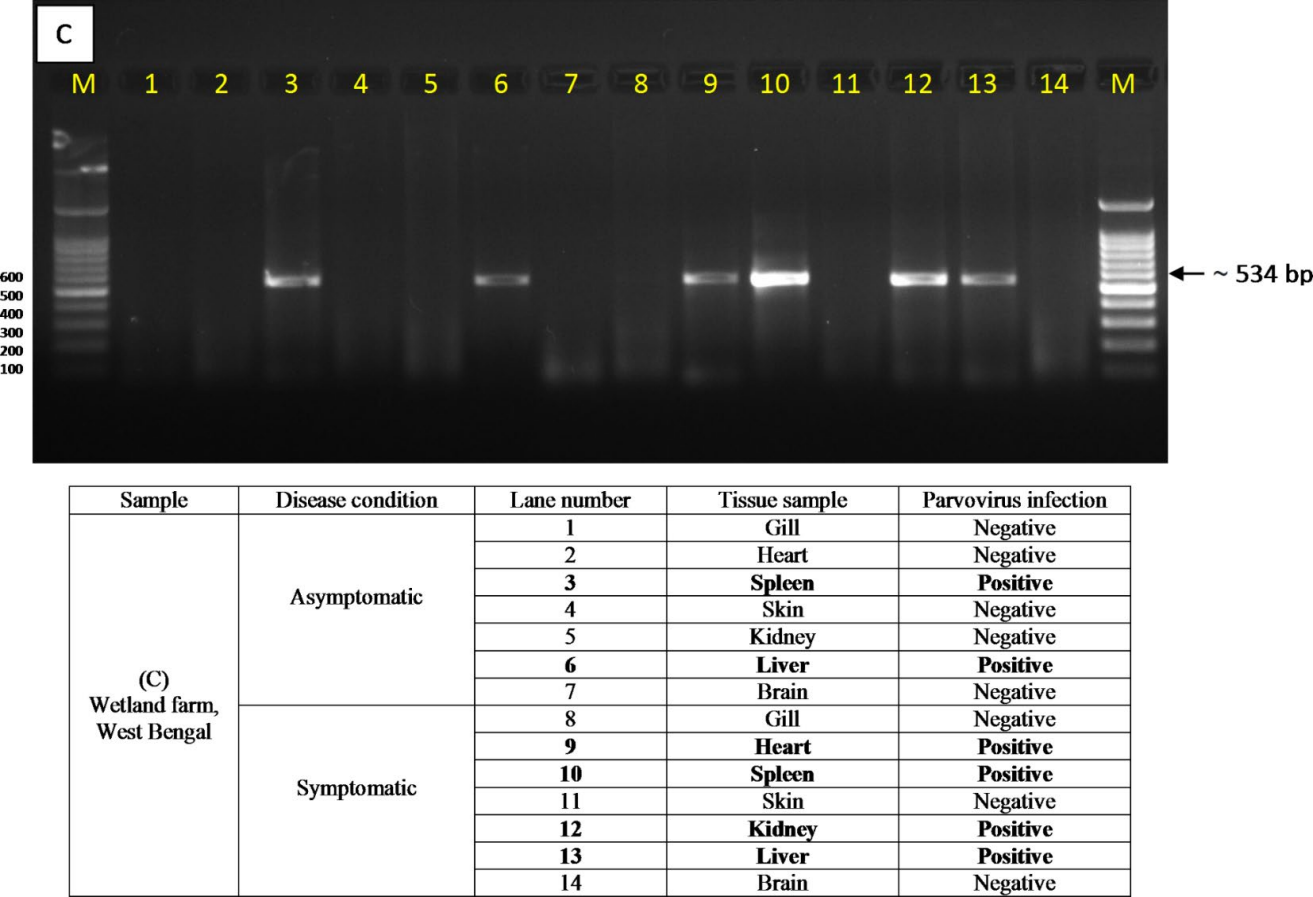
Agarose gel of PCR amplicon from symptomatic and asymptomatic tilapia tissue samples using TiPV specific primer. (A) Tissue samples were collected from a Wetland farm, Orissa; (B) Samples from a cage farm, Orissa; and (C) tissue samples from a wetland farm, West Bengal. M- 100 bp DNA ladder; Lane 1- Gill, 2- Heart; 3- Spleen; 4- Skin; 5- Kidney; 6- Liver and 7- Brain. Positive amplicon (~ 534 bp) exhibits TiPV positive template DNA.

Considering the involvement of a novel TiPV viral pathogen’s role in massive mortality, we collected the tissue samples for molecular characterization and cell line validation. PCR amplification and detection of tilapia-parvovirus from heart, liver, spleen, kidney, gill, brain and skin tissue samples of symptomatic and asymptomatic tilapia samples collected from wetland and cage farms. Positive results for TiPV were observed in gill, heart, spleen, liver and kidney samples collected from symptomatic tilapia samples from cage and wetland farms. In contrast, negative results were found in the brain and skin tissue samples (Fig. 2A, B). In contrast, only the heart, liver and spleen showed positive results for TiPV in asymptomatic tilapia tissue samples. Notably, the spleen displayed the best results for screening TiPV diagnosis. Interestingly, the presence of TiPV in asymptomatic samples suggests its widespread dispersion and possible synergistic effects in existing microbial infections.

### Sequencing and phylogenetic analysis

**Fig. 3:**
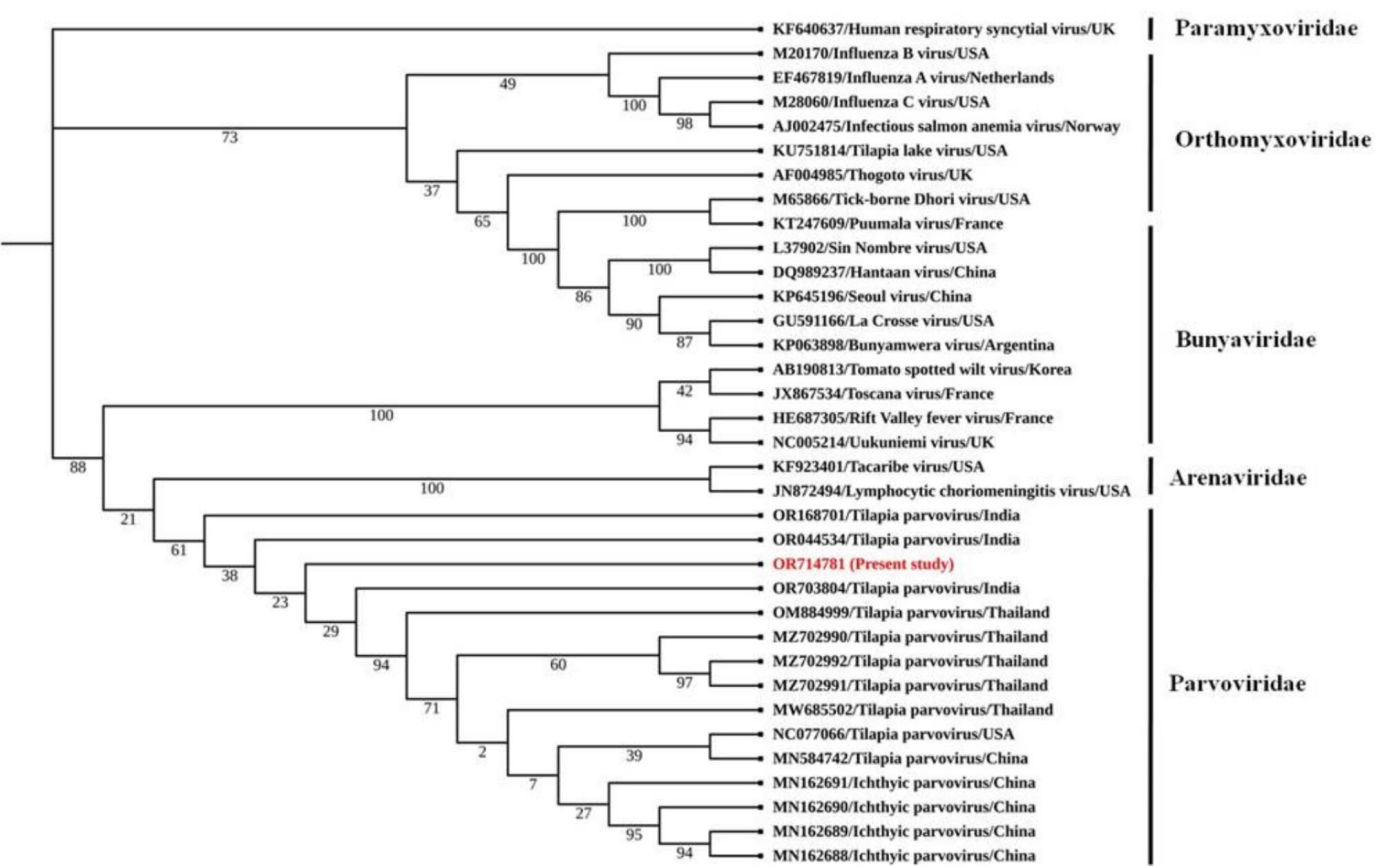
Phylogenetic tree analysis of TiPV isolate based on nucleotide sequences following maximum composite likelihood method by the MEGA11 software. The numbers next to the branches indicate percentage values for 1000 bootstrap replicates. Bootstrap values above 50% are shown at the nodes. The isolate was categorized into 3 clusters indicated by a shad of different colour.

Nucleotide BLAST of the partial sequences revealed that the isolated virus species was TiPV. The sequence was submitted to GenBank, and accession number OR714781 was obtained. The results showed that isolated TiPV has > 99% similarity with other reported TiPV sequences (MW685502, OR044534, OM884999 and MZ702991). Multiple sequence alignment (CLUSTALW) of the partial sequence revealed that sequences of recovered TiPV were identical and showed maximum similarity with TiPV reported from India, Thailand and China. Based on their relatedness, all of the Indian-TiPVs were shown to belong to a single evolutionary clade in the phylogenetic tree. Moreover, based on sequence homology, it can be inferred that isolated TiPV is more closely related to the other two reported Indian-TiPV than the TiPV originated from Thailand and China (Fig. 3).

### Virus isolation in cell line

**Fig. 4:**
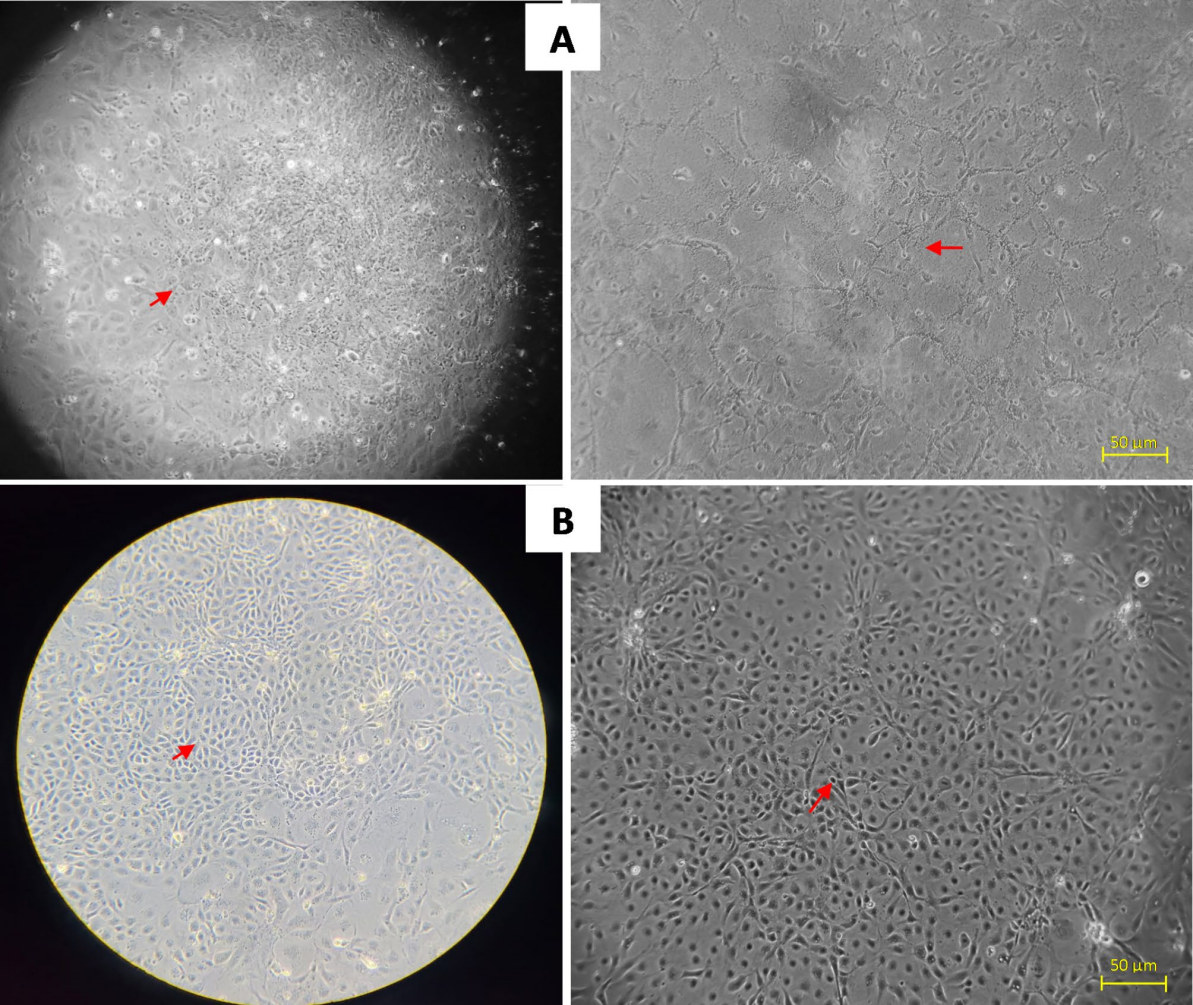
(A) Infection of DRG cell line (developed from *Danio rerio* gill) with filtered tissue homogenate prepared from spleen of TiPV PCR positive tilapia sample; (B) Control DRG cell line without homogenate inoculation.

On the initial isolation, tissue homogenates were inoculated onto the DRG cell line. Cell morphology changed as early as two days post-infection (dpi), and cytopathic effect (CPE) was observed at six dpi. Cytoplasmic vacuolization, rounding, and shrinking of cells were common CPEs. Eight dpi completely destroyed the cell monolayer, with more than 60% of the cells detached (Fig. 4). The PCR of DNA samples from the cell pellets yielded a TiPV-specific positive amplicon.

### Histopathological changes

**Fig. 5:**
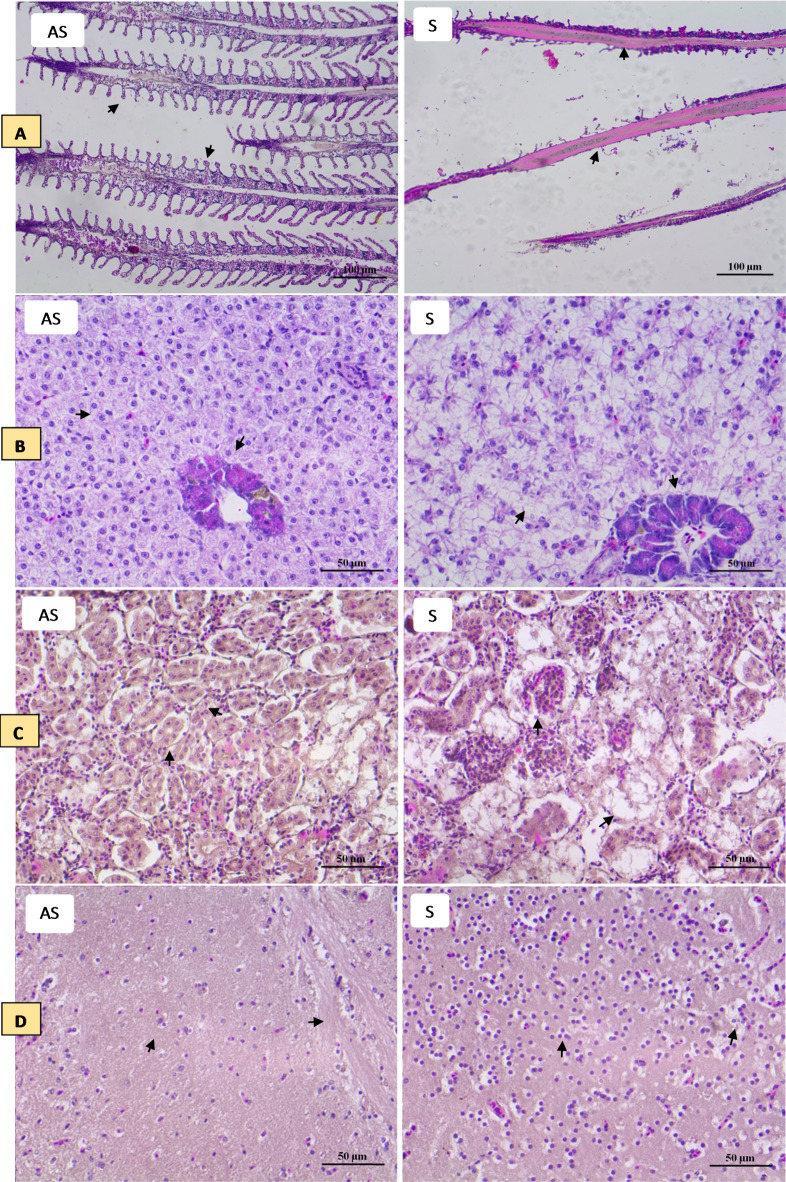

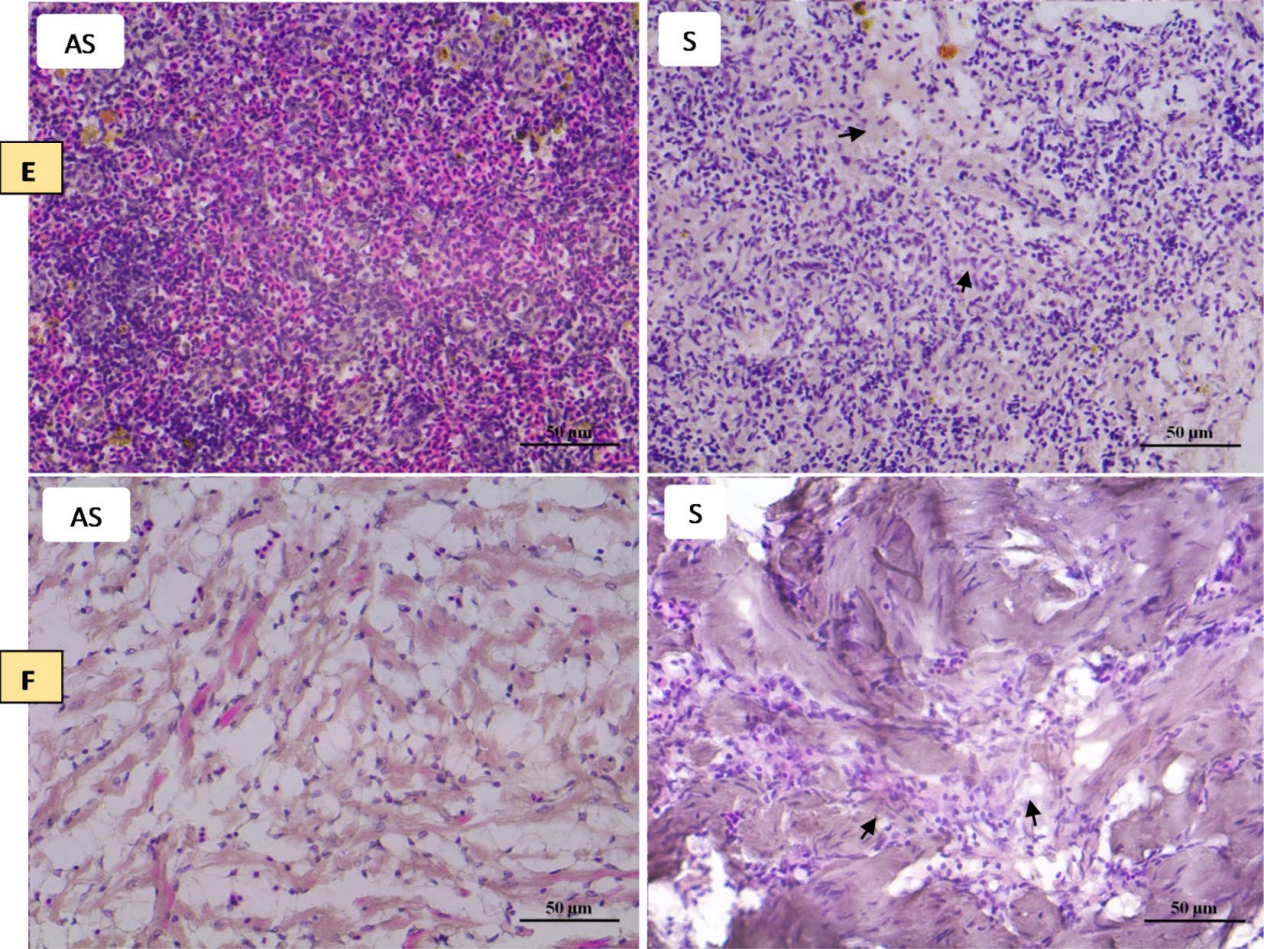
Microphotographs of histological section (H&E) from (A) gill, (B) liver, (C) kidney, (D) brain, (E) spleen, and (F) heart samples of tilapia samples. (AS) asymptomatic tilapia samples and (S) symptomatic tilapia samples. The arrowhead in figures (A–F) represents the modulation in tissue morphology of fish during Tilapia parvovirus infection. In gills (S), epithelial hyperplasia, curling of secondary lamellae, aneurism, epithelial lifting, dilation, and congestion in the blood vessels, and thickening and clubbing of the secondary and primary lamellae engorged with blood cells. In liver (S), the hepatic cells were denatured and necrotic, and the nucleus and cytoplasm were concentrated. Some hepatic cells were vacuolated and had dissolved nuclei. In the Kidney (S), renal tissue was disorganized, with swelling and degeneration of cells, a narrow renal cavity, and a transparent tube type. In brain (S), degenerations, vacuolar degeneration, edema, necrosis, hemorrhage, and congestion in the blood vessels. In spleen (S), atrophy of the white pulp, and a reduced area occupied by splenic pulp hyperemia were noted. In heart (S), epicardial infiltration with lymphocytes, plasma cells, and macrophages and degeneration of the coronary blood vessel, the area with unaltered blood vessels, and epicardium.

To observe the histopathological conditions of tilapia symptomatic with TiPV, HE staining was performed using tissues from the gill, liver, kidney, brain, spleen and heart samples from symptomatic and asymptomatic tilapia samples (Fig. 5). Normal primary and secondary gill lamellae were seen in the control group (Fig. ​5 A NI). Meanwhile, sloughed epithelial cells of secondary lamellae were seen in most examined gills in symptomatic fish. Epithelial hyperplasia, curling of secondary lamellae, aneurism, epithelial lifting, dilation, and congestion in the blood vessels, and thickening and clubbing of the secondary and primary lamellae engorged with blood cells were also observed (Fig. ​5 A I). In the asymptomatic group, the hepatic cells were rich in the cytoplasm, had a prominent nucleus and cytoplasm, had a clear cell boundary, and were evenly dispersed (Fig. 5B NI). In the meantime, inflammatory cells in the liver of tilapia exhibiting symptoms displayed localized aggregation with a vague border and uncertain organization. The cytoplasm and nucleus were concentrated within the necrotic, denatured liver cells. There were some vacuolated liver cells with dissolved nuclei. There was necrosis and degeneration near the sinuses, and the tissue was loose and oedematous. Additionally, the tissue structure was destroyed, and numerous hepatocytes were vacuoted (Fig. 5B I). The symptomatic tilapia exhibited a disorganized renal structure consisting of a limited renal cavity, a clear tube type, and cell swelling and degeneration. There were several bleeding areas, lymphocytes throughout the renal tissue, and what appeared to be an inflammatory response (Fig. 5C I). Histopathological changes appeared in the brain tissue of symptomatic tilapia samples, including degenerations, vacuolar degeneration, edema, necrosis, hemorrhage, and congestion in the blood vessels (Fig. ​5D I). The usual red pulp structure with normally compacted ellipsoids was visible in the splenic portion of the control fish (Fig. 5E NI). Additionally, in symptomatic tilapia, ferriflavin accumulation in the splenic tissue, atrophy of the white pulp, and a decreased area occupied by splenic pulp hyperemia was observed (Fig. 5E I). Fish that were symptomatic displayed degeneration of the coronary blood vessel, the region with unchanged blood arteries, and epicardium, as well as epicardial infiltration with lymphocytes, plasma cells, and macrophages. Furthermore, fish that had been exposed showed oedematous intracellular vacuolization of the heart (Fig. ​5 F I).

### Quantification of TiPV distribution and viral loading in different tissues

**Fig. 6:**
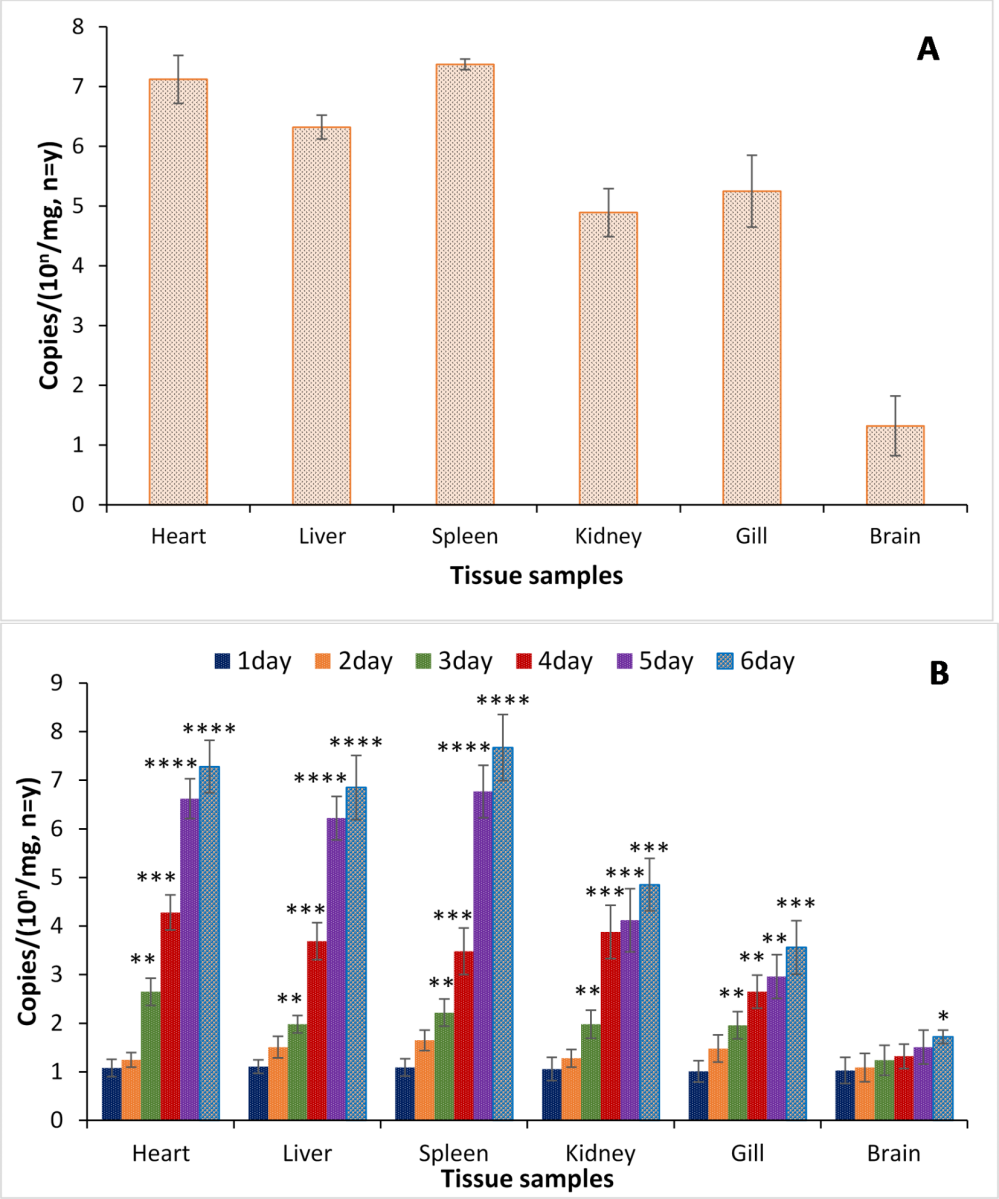
Genome copies and distribution of TiPV in symptomatic tilapia tissue samples. (A) qPCR assay of TiPV genome copies in different tissues of the symptomatic tilapia samples. (B) TiPV genome copies in different symptomatic tilapia tissue samples compared to asymptomatic samples at different time points (1, 2, 3, 4, and 5 days post-infection). The results are the mean ± SE (*n* = 3) and the vertical bars with asterisk indicate significant differences between treatment groups (*P* < 0.05).

The symptomatic tilapia’s heart, liver, spleen, kidney, gill, brain, and skin tissue were taken, and quantitative real-time PCR was used to find viral genome copies in these samples. The genome copies of the virus in the symptomatic liver (3.1 × 10^6.32 ± 0.21^/mg), spleen (3.9 × 10^7.37 ± 0.35^/mg), and heart (3.3 × 10^7.12 ± 0.36^/mg) were higher than those from the gill (4.2 × 10^5.25 ± 0.27^/mg), or kidney (2.2 × 10^4.89 ± 0.24^/mg). In brain samples, genome copies of the virus were lower than above tissues and showed genome copies number of 1.8 × 10^2.32 ± 0.17^/mg. Moreover, no significant viral quantities were found in muscle tissue samples of tilapia (Fig. 6A).

Quantitative real-time PCR of TiPV was also performed to estimate viral loads in the six tilapia tissues (gill, heart, spleen, liver, kidney and brain) sampled at various time points up to 6 days post-TiPV challenge. Study showed that DNA of the virus was detected in the examined tissues of the kidney and spleen at 1 dpi and then continued to increase by 5 dpi in the TiPV-challenged fish. In contrast to the spleen and kidney tissues, the virus content in the heart, gut, and brain tissues increased steadily between 1 and 3 dpi before peaking at 6 dpi. Gill, liver, and ocular tissue had lower TiPV levels up until 3 dpi, but at 6 dpi, maximum values were found (Fig. 6B).

### Effect of TiPV infection on tilapia immune response

**Fig. 7:**
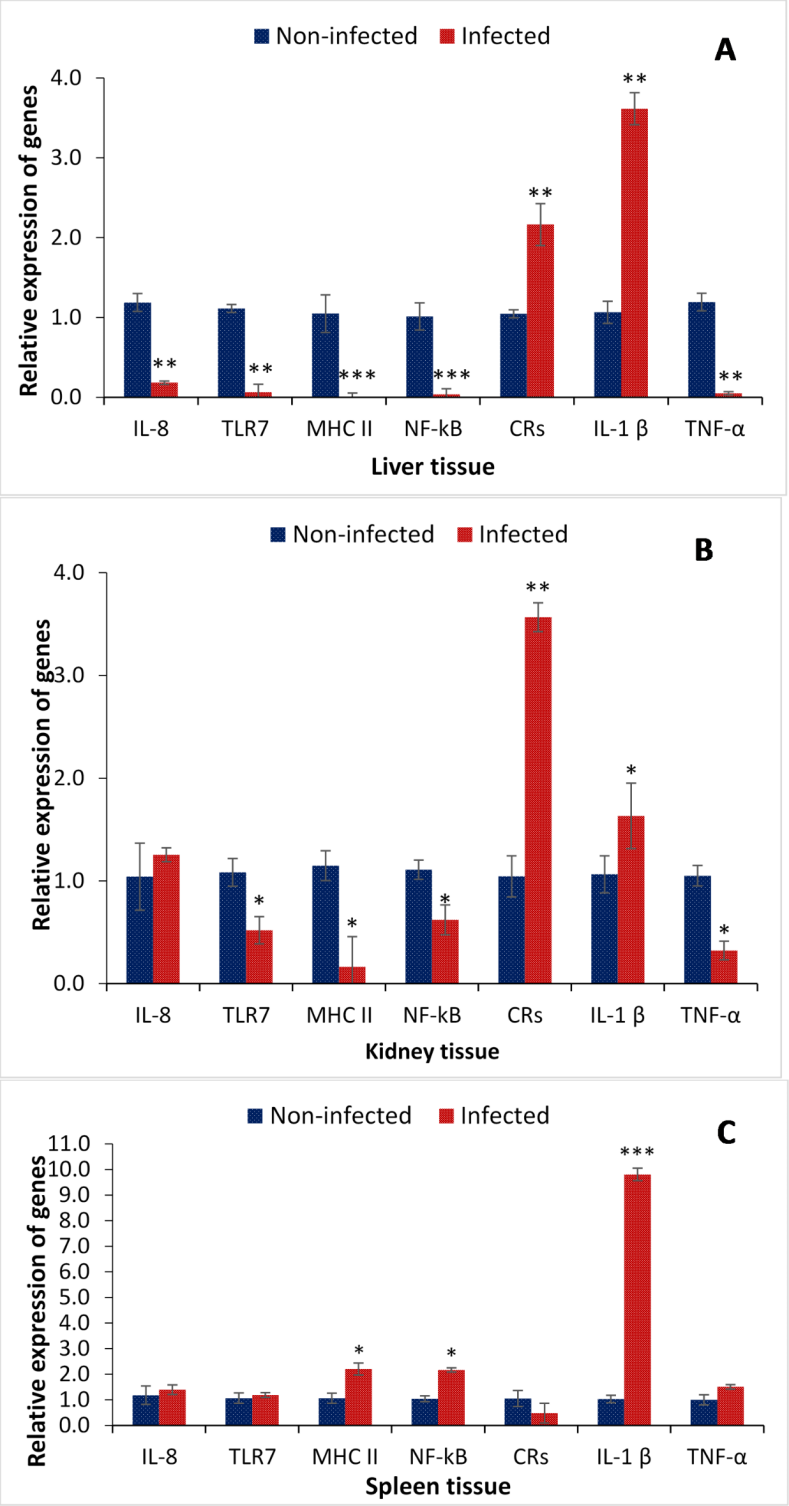
Fold change in gene expression of tilapia tissue samples in asymptomatic and symptomatic groups. Expression of Interleukin-8 (*IL-8*), Toll-like receptors (*TLR7*), major histocompatibility complex (*MHC-II*), Nuclear factor kappa B (*NF-κB*), chemokine receptors (*CRs*), Interleukin-1 (*IL-1β*) and Tumour necrosis factor α (*TNF-α*) genes by quantitative real-time PCR. The temporal expression was analyzed from asymptomatic and symptomatic tilapia from (A) liver, (B) Kidney, and (C) spleen tissue samples. The expression level in the asymptomatic group was regarded as 1.0, and thereby, the expression ratio of the symptomatic group was expressed as compared to the control group. The results are the mean ± SE (*n* = 3), and the vertical bars with an asterisk indicate significant differences between treatment groups (*P* < 0.05).

As there is a correlation between viral pathogenesis and fish health, the in vivo temporal difference in the expression of Interleukins (*IL8*, *IL1β*), Toll-like receptors (*TLR7*), major histocompatibility complex (*MHC-II*), Nuclear factor kappa B (*NF-κB*), chemokine receptors (*CRs*) and Tumour necrosis factor α (*TNF-α*) genes was investigated at the transcriptional level. The results showed that the health condition of tilapia is immunocompromised during Tilapia Parvovirus infection. The studied immune-related genes, including *IL-8*, *TLR7*, *MHC-II*, *NF-κB*, and *TNF-α*, were significantly downregulated in liver and kidney tissues. Upregulation or higher transcription values were recorded for *CRs* (~ 2 and ~ 4 folds) and *IL-1β* (~ 4 and ~ 2 folds) in liver and kidney tissue, respectively (Fig. 7A, B). Moreover, the *MHC II*, *NF-kB* (~ 2 folds), and *IL-1β* (~ 10 folds) gene expressions were increased significantly in the symptomatic tilapia spleen samples (Fig. 7C). In contrast, the transcription of *IL-8*, *TLR7*, *CRs*, and *TNF-α* was significantly downregulated in the symptomatic group compared with the asymptomatic control tilapia samples. Although there are variations in the expression profile of immune-related genes, the results suggest a significant interplay between TiPV infection and immune components. The virus might downregulate immune cells to establish itself in the host and vice versa, which needs further validation.

### Virulence of TiPV on O. Niloticus

**Fig. 8:**
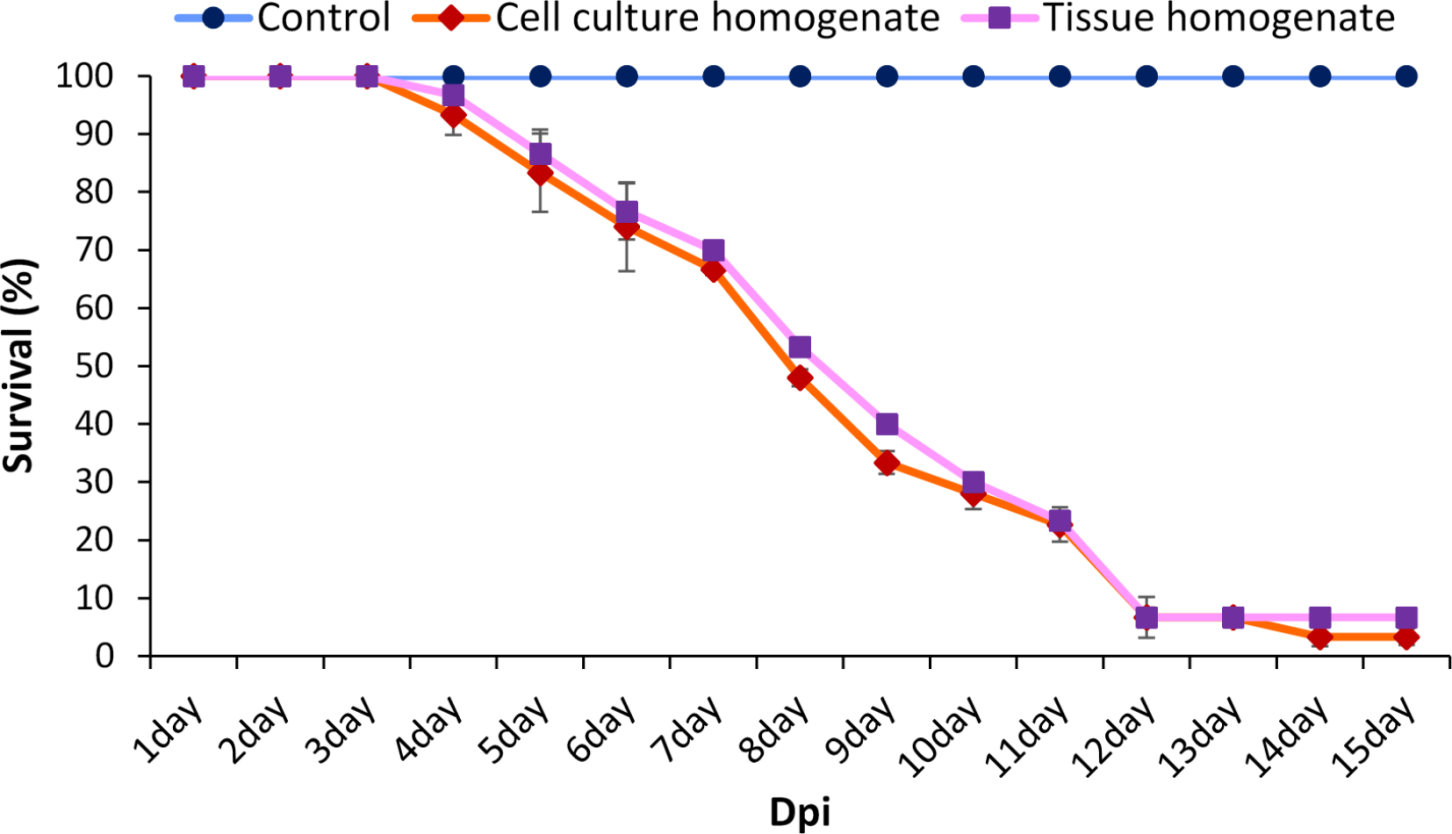
Survival assay of *O. niloticus* challenged with cell culture viral supernatant and symptomatic fish tissue homogenate supernatant. The fish were intraperitoneally injected with (A) cell culture viral supernatant (10^4.0^ TCID_50_/ml), (B) 50 µl of symptomatic fish tissue homogenate filtrated supernatant, and (C) a control group with 50 µl of sterile Phosphate-buffered saline (PBS). The results are the mean ± SE (*n* = 3).

The tilapia challenged with cell culture homogenate or symptomatic fish sample homogenate exhibited clinical lesions and hemorrhagic symptoms similar to those found in naturally diseased fish at ~ 4–6 days post-infection. Fish mortality was maximum at 92% within 12 days post-infection (Fig. 8). In parallel with cell culture homogenate injection symptoms and mortality, the symptomatic fish sample homogenate induced a similar effect on infected tilapia samples. The samples of moribund or dead challenged or control group tilapia were collected and processed to determine the presence of TiPV in the samples. Results showed that moribund or dead samples in the challenged group were TiPV positive and confirmed that it was indeed the etiologic agent of the observed disease in the tilapia farms.

## Discussion

Over the past thirty years, the worldwide demand for tilapia has significantly contributed to the growth of tilapia aquaculture^35^. Nonetheless, the global expansion of tilapia farming, along with frequent movement of live fish and their products between countries and the impact of climate change, may lead to the emergence and spread of new diseases affecting aquaculture^11,36^. Recently, diseases in farmed tilapia have spread globally, including in India, causing mass mortality outbreaks and impacting production. These diseases include Tilapia Lake Virus (TiLV) and Infectious Spleen and Kidney Necrosis Virus (ISKNV)^37–39^. Another emerging disease in farmed Tilapia is Tilapia Parvovirus (TiPV), which has been reported in several aquaculture-producing nations (TiPV)^8^. This virus, initially identified in bait Nile tilapia, has caused significant mortality rates and severe economic losses to tilapia aquaculture^22^. In this study, we investigated TiPV infection at multiple tilapia farms in India. The study characterized novel TiPV associated with mass mortality in wetland and cage farms of tilapia. This novel viral pathogen was characterized following an investigation of clinical symptoms, molecular and phylogenetic analysis, cell line assay, pathological changes, viral distribution and quantification analysis, and viral-mediated host immune response.

In this research, we identified the recently described tilapia pathogen TiPV through genetic and phylogenetic characterization along with clinical and pathological presentation. TiPV has been detected from different life stages, including fingerlings to adult-size fish from floating cages and wetland tilapia farms. Fish being exposed to a rich microbiome and concomitant stressors in their aquatic environment, involvement of multiple microbial agents with bacteria, parasites and viruses (TiLV) are common in aquaculture and often occurring during natural disease outbreaks^40–45^. However, we did not find fish being infected with other viral (e.g., TiLV) or any opportunistic pathogens, and thus, it is possible that TiPV was a potential causative agent for mass morality in tilapia farms. However, infected fish become highly immunocompromised, making them more susceptible to environmental opportunistic pathogens. As farmers source fish from various locations and raise infected tilapia in different water bodies like wetlands and reservoirs, tracking the origins of disease outbreaks is quite challenging. Many factors can contribute to the spreading of aquatic viruses; these include live fish movement, natural carriers, water-shedding, or contamination through humans or fomites. Furthermore, stress or compromised immunity in fish can make them more vulnerable to various pathogens, including these two viruses. Additional studies are needed to confirm the source of the infection. A high prevalence of TiPV was detected in apparently healthy tilapia collected from tilapia cage and wetland farms of Orissa and West Bengal. Tilapia Parvovirus was detectable in gill, heart, spleen, liver, and kidney samples collected from tilapia samples. The highest TiPV burdens were found in the spleen, liver, and heart compared with other organs, suggesting that spleen is mainly targeted by Tilapia parvovirus and would be an ideal tissue for diagnostics purposes. Moreover, the presence of TiPV in asymptomatic samples suggests its wide distribution and potential synergistic effects in existing microbial infections; however, more studies are needed to confirm this observation. Tilapia Parvovirus was recently detected in the various organs of Nile tilapia in China, India and red hybrid tilapia in Thailand^6,8,19^. Therefore, understanding TiPV’s host range and genomic variation is necessary to gain better insights into farm-level risk factors and develop appropriate management strategies.

Aquatic organisms are constantly exposed to microbial pathogens; therefore, non-specific and specific immune factors could potentially serve as preventive measures against biotic and abiotic stressors in aquaculture farming^46,47^. However, microbial pathogens have developed several methods, through the expression of toxins or virulence factors, to mask the cascade of diverse immune responses of fish aiming to eliminate the recognized foreign agent and restore homeostasis^30–32,48–50^. Hence, it’s become essential to understand the fish immune response during microbial infection in order to identify key immune regulators for developing management strategies.

The expression of immune-related genes (e.g., Interleukins (*IL8*, *IL1β*), Toll-like receptors (*TLR7*), major histocompatibility complex (*MHC-II*), Nuclear factor kappa B (*NF-κB*), chemokine receptors (*CRs*) and Tumour necrosis factor α (*TNF-α*)) is usually considered as a sign for immune stimulation or enhanced immune response^51–53^. Interleukin (*IL8*, *IL1β*) and Tumour necrosis factor α (*TNF-α*), a classic pro-inflammatory cytokine, play a central role in early inflammatory response by mediating immediate and vigorous response, inducing several inflammatory reactions^29,54,55^. The Toll-like receptors (*TLRs*), major histocompatibility complex (*MHC*) and chemokine receptors (*CRs*) are a significant class of receptors that make up the immune system’s initial line of defence against microorganisms. They are essential in bridging innate and adaptive immunity because they can identify both invasive infections and endogenous danger compounds generated from injured and dying cells^56–58^. The transcription factor nuclear factor-κB (*NF-κB*) plays a critical role in mediating responses to a remarkable diversity of external stimuli and, thus, is a pivotal element in multiple physiological and pathological processes and is a powerful orchestrator of the immune response^59,60^. Moreover, in severe infections caused by microbial pathogens, viz., viruses, animals have general health degradation and eventually die due to the immune system’s inability to handle pathogen-mediated cellular damage. Furthermore, several microbial pathogens, such as viruses, have developed strategies for modifying immune response activity to infiltrate the host and evade the strong immune response. Hence, it becomes important to understand immunity during virus-mediated disease outbreaks to understand host and pathogen interactions and develop therapeutic measures and management guidelines. During TiPV infection, significant downregulation of *IL-8*, *TLR7* and *TNF-α* were observed in tilapia tissue samples. While upregulation of *CRs*, *IL-1β*, *MHC I*I, and *NF-kB* was recorded in symptomatic tilapia samples. The gene expression analysis corroborated that TiPV severely impacts the host’s immune response; however, upregulation of certain immune genes is possibly an effort from the host response to protect the animal from invading viral pathogens.

The collected tilapia samples from wetlands and cage farms exhibited similar clinical signs and symptoms reported in naturally TiPV-infected tilapia samples. This suggests that TiPV is an emerging disease and may have spread across various tilapia farms, irrespective of culture system. Although TiPV, the family Parvoviridae, have very controlled host ranges and tissue tropisms, we could able to grow the TiPV in the *Danio rerio* (Zebrafish) cell line to serve for isolation of the virus and study the viral pathogenesis. The virus can propagate in this cell line, which allows us to harvest and challenge pathogens by injecting them into healthy fish. The resulting disease manifested, and clinical signs matched those of naturally infected tilapia. The study is helpful in confirming that TiPV can be isolated in *Danio rerio* cell lines and induce significant mortality in tilapia, confirming the etiologic agent of this disease.

In conclusion, the study revealed the widespread occurrence of Tilapia Parvovirus (TiPV) in cage and wetland aquaculture systems in India. The virus was detected in healthy and diseased fish, indicating the virus may have a wider geographical distribution and may spread fast through the transboundary movement of tilapia. Apart from typical clinical conditions, the virus induces significant pathological and immunological modulation in the host, which might be responsible for high mortality in tilapia aquaculture. Since there are several reports of multiple microbial co-infections with TiPV, further studies to evaluate the severity of disease in the presence of microbial pathogens need to be evaluated for developing a holistic approach to TiPV management. In addition, extensive work on screening TiPV in non-tilapia species is needed to establish the virus’s geographical distribution, prevalence, and spread of the virus.

## Electronic supplementary material

Below is the link to the electronic supplementary material.

Supplementary Material 1

## Data Availability

The datasets generated and/or analyzed during the current study are available in the NCBI repository, accession number OR714781.
