# Supplementary material for: Pathological effects and immune modulation in host during Tilapia Parvovirus (TiPV) outbreak in cage and wetland Tilapia farms
Source: Sci Rep. 2024 Nov 20;14:28689. doi: 10.1038/s41598-024-79089-5 (PMC11577022; doi:10.1038/s41598-024-79089-5)
Supplement: Supplementary file 1 — Supplementary Material 1 [file 41598_2024_79089_MOESM1_ESM.docx]

S1: Agarose gel of PCR amplicon from symptomatic and asymptomatic tilapia tissue samples using TiPV-specific primer

M 1 2 3 4 5 6 7 8 9 10 11 12 13 14 M


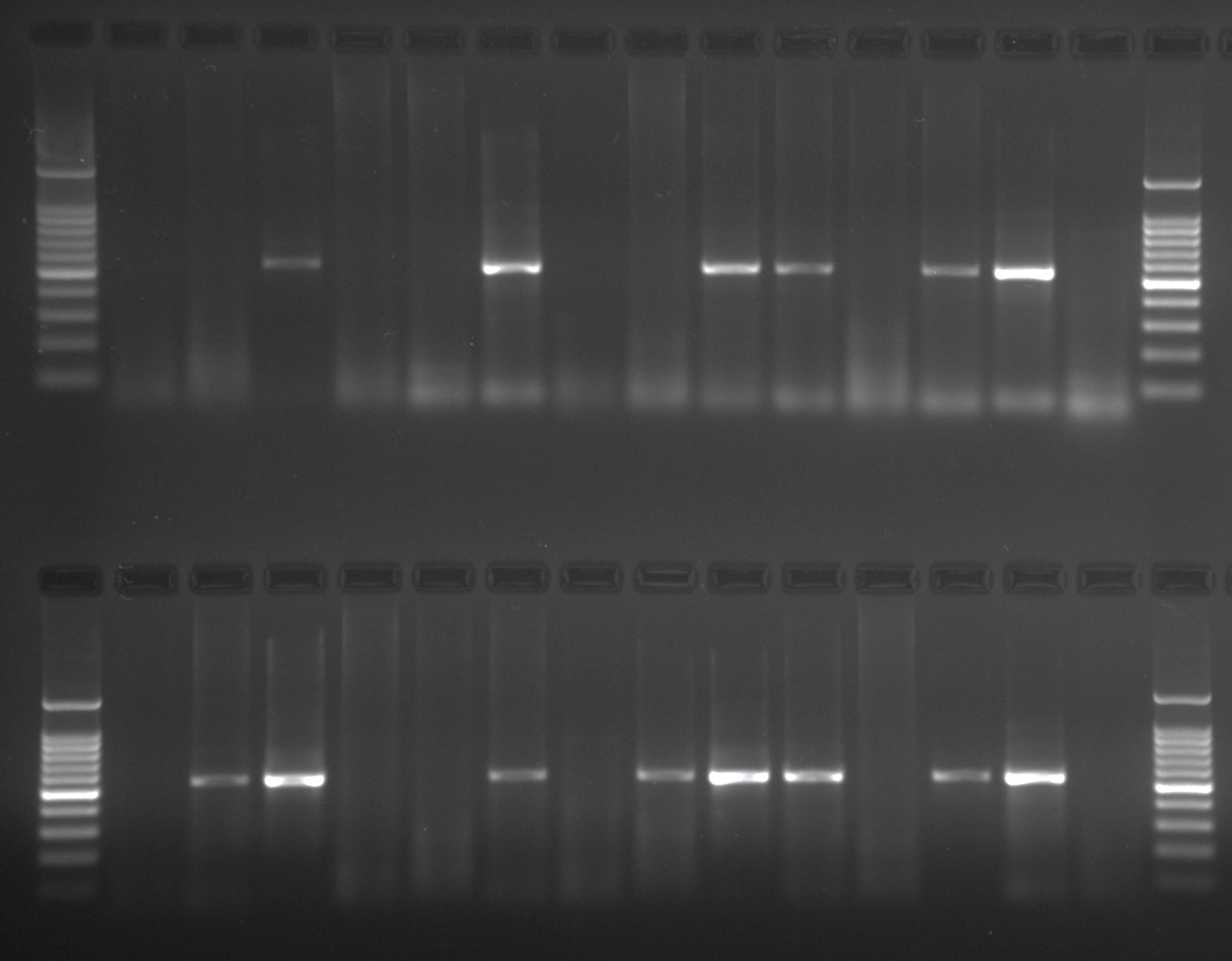


B

A

M 1 2 3 4 5 6 7 8 9 10 11 12 13 14 M


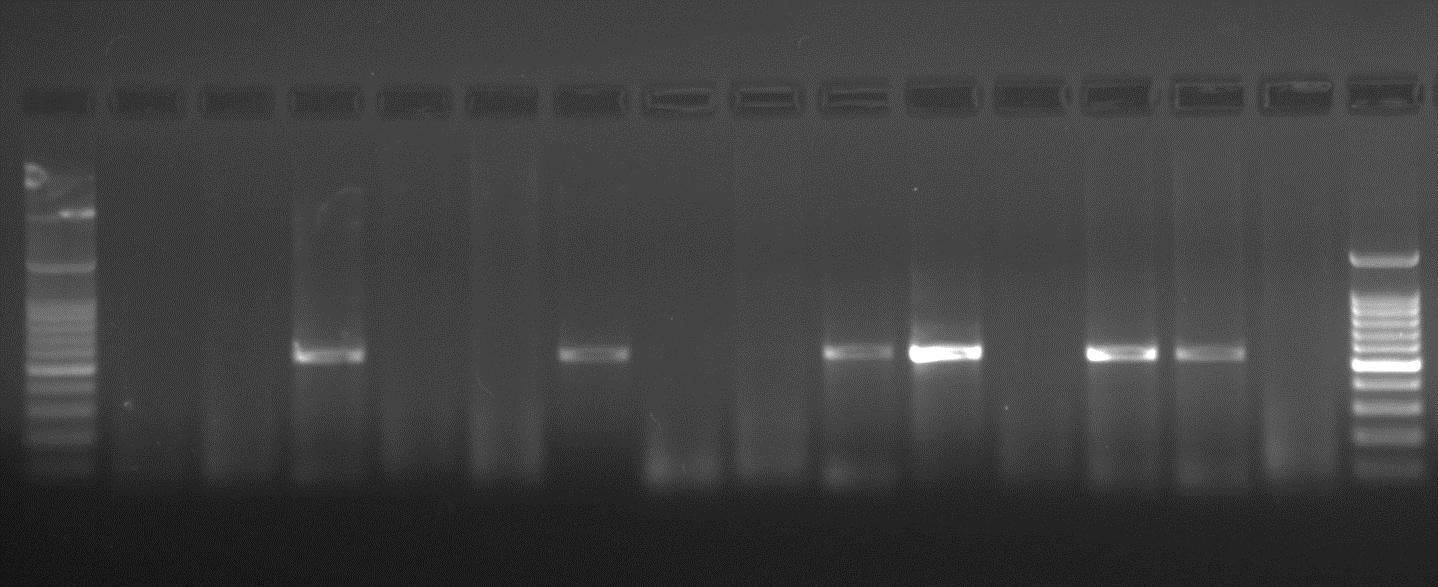


C
